# The ctenophore *Mnemiopsis leidyi* regulates egg production via conspecific communication

**DOI:** 10.1186/s12898-018-0169-9

**Published:** 2018-03-26

**Authors:** Daniel A. Sasson, Anya A. Jacquez, Joseph F. Ryan

**Affiliations:** 1 Whitney Laboratory for Marine Bioscience, University of Florida, St. Augustine, FL USA; 2 Department of Biology, Saint Louis University, Saint Louis, MO USA; 3 Department of Biology, Lewis & Clark College, Portland, OR USA; 4 Department of Biology, University of Florida, Gainesville, FL USA

**Keywords:** Hermaphrodite, Self-fertilization, Egg-trading, *Mnemiopsis leidyi*, Reproduction

## Abstract

**Background:**

Communication between individuals of the same species is an important aspect of mating and reproduction in most animals. In simultaneously hermaphroditic species with the ability to self-fertilize, communication with conspecifics can be essential to avoid inbreeding depression. One such behavioral adaptation observed in some simultaneous hermaphrodites is gamete trading. This behavior involves individual hermaphrodites in pairs alternating between reproducing as the male and female, and, as such, necessarily requires communication and coordination between mates. Little is known about communication in ctenophores and conspecific communication has not been described in this group; however, our previous work suggested that the ctenophore *Mnemiopsis leidyi* might engage in gamete trading. We tested for this possibility by constructing divided arenas (both sealed and permeable) that allowed us to measure individual egg output for paired *M. leidyi*.

**Results:**

We found that, when not allowed to interact, size-matched individuals produced similar numbers of eggs on each side of the arena. However, if allowed to interact and exchange water, size-matched pairs produce significantly different numbers of eggs on each side of the arena, suggesting that these pairs use chemical communication to modulate reproduction in the presence of conspecifics as would be expected in gamete trading.

**Conclusion:**

This finding presents exciting new possibilities for future investigations into the nature of signaling in *M. leidyi*. Furthermore, this first evidence of conspecific communication in Ctenophora, a group that branched off from the rest of animals more than 600 million years ago, has significant implications for the signaling ability of the last common ancestor of all animals.

**Electronic supplementary material:**

The online version of this article (10.1186/s12898-018-0169-9) contains supplementary material, which is available to authorized users.

## Background

Signaling between individuals within a species is common in animals and is used for a wide variety of functions, such as habitat selection [1, 2], predator avoidance [3, 4], and mate attraction and acquisition [5]. The majority of what is known about signaling between conspecifics has focused on visual and/or auditory communication [6]; however, chemical communication is pervasive and is presumably the main form of signaling in many animals. Little to nothing is known about conspecific communication in ctenophores (comb jellies). With growing evidence suggesting that Ctenophora is the sister lineage to the rest of animals ([7–16] but see [17, 18]), investigating the nature of conspecific signaling in ctenophores is essential to our understanding of the role of signaling in the earliest animals and throughout animal evolution.

Conspecific communication is fundamental to animal reproduction. Some of the most elaborate traits in animals, such as bird song or fiddler crab claws, are common signals used by males to attract females or dominate rivals [5]. While pervasive in animals with separate sexes, these sexually dimorphic traits are not found in species with simultaneous hermaphroditism as all individuals are both male and female. Yet, reproductive signaling is almost certainly just as important in these animals. For example, externally fertilizing simultaneous hermaphrodites face behavioral challenges related to self-fertilization. Self-fertilization can be advantageous, especially in the absence of mates, but reduced offspring fitness due to inbreeding depression may be a major drawback [19, 20]. Thus, there may be strong selection for physiological (e.g. sperm/egg incompatibility) or behavioral mechanisms to avoid inbreeding depression in hermaphrodites capable of self-fertilization.

One behavioral mechanism simultaneously hermaphroditic animals may employ to avoid self-fertilization is gamete trading [21]. Gamete trading involves mating pairs reciprocally alternating between male and female roles during successive mating bouts. This behavior is generally thought to be an adaptation to ensure the availability of both male and female gametes during mating while also preventing one individual from being saddled with all of the costs associated with female gamete production [21]. For externally fertilizing organisms, this behavior may also ensure that self-fertilization does not occur [22]. Gamete trading has been described in a number of simultaneous hermaphroditic animals, including polychaetes [23], sea slugs [21, 24], and fish [22, 25]. The mechanism by which gamete trading occurs is unknown in most species, but this behavior likely requires communication between mating pairs to establish spawning order. Somewhat surprisingly, gamete trading has never been described in non-bilaterian animals (i.e., Ctenophora, Porifera, Placozoa, and Cnidaria), despite the large numbers of species in these clades that are simultaneous hermaphrodites [26].

The ctenophore *Mnemiopsis leidyi* is a gelatinous marine predator of zooplankton native to the Atlantic coast of North and South America. *Mnemiopsis leidyi* is known for its role as an invasive species, having reached Europe through transport in the ballast water of oceanic cargo ships [27]. Like most ctenophores, *M. leidyi* is a simultaneous hermaphrodite with the ability to self-fertilize [28]. Sperm are released into the water column followed by eggs that are then fertilized [28]. Self-fertilization has been proposed as the primary method through which most ctenophores reproduce [28], and much of *M. leidyi’s* success in establishing in non-native waters may be due to its ability to self-fertilize [29]. However, recent work has shown that self-fertilization can be costly; *M. leidyi* spawned in isolation have lower offspring viability than *M. leidyi* spawned in pairs, possibly due to inbreeding depression [30]. Given that self-fertilization is generally costlier than out-crossing, it would stand to reason that ctenophores might have evolved physiological or behavioral mechanisms to minimize self-fertilization when in the presence of conspecifics.

Behavioral avoidance of self-fertilization would require *M. leidyi* to detect conspecifics, and currently the only evidence suggesting this ability in ctenophores is indirect. For example, *M. leidyi* is able to detect and respond to the predatory ctenophore *Beroe ovata* [31]. Likewise, *Beroe cucumis* increases swimming activity when exposed to water conditioned by *Bolinopsis infundibulum*, suggesting that *B. cucumis* can detect its ctenophore prey [32]. Histological and developmental studies have further suggested the presence of chemoreceptor cells in the lips of beroid ctenophore species [33, 34]. These studies provide evidence that ctenophores can detect chemical cues produced by heterospecific species. However, to our knowledge, no study to date has reported that ctenophores use chemical cues for anything other than predator/prey interactions nor that ctenophores can detect chemical cues secreted by conspecifics.

Our previous study on inbreeding depression provided tantalizing evidence that *M. leidyi* may detect the presence of conspecifics. Paired *M. leidyi* spawned the same total number of eggs as isolated individuals [30], suggesting the possibility that only one of the paired *M. leidyi* spawned eggs. These results suggest *M. leidyi* may not only be able to detect conspecifics but also alter their reproductive behavior in the presence of other individuals. In this study, we evaluate the hypothesis that *M. leidyi* communicates with conspecifics to modulate spawning behavior.

## Methods

### Aquaria design

**Fig. 1:**
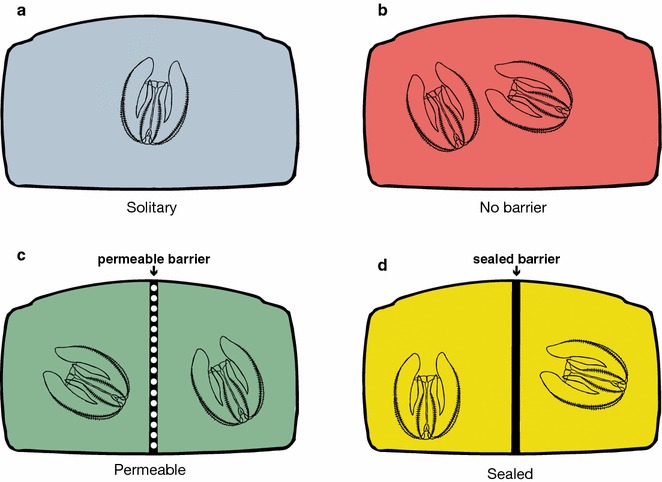
Experimental design. **a** Solitary treatment: one *M. leidyi* in an arena without a barrier. **b** No-barrier treatment: two *M. leidyi* in an arena without a barrier. **c** Permeable-barrier treatment: two *M. leidyi* in an arena separated by a perforated barrier. **d** Sealed-barrier treatment: two *M. leidyi* in an arena separated by a barrier without holes

We created experimental arenas to test for gamete trading by modifying plastic Aqueon Betta Bowl Aquaria (model #100101216), which come with an acrylic divider. We sawed the tops of these tanks so that each was 15 cm tall. We created three types of arenas: (1) sealed-barrier arenas, (2) permeable-barrier arenas, and (3) no-barrier arenas (Fig. 1). For the sealed-barrier arenas, we sealed the existing large holes and the edges of the divider to the arena with marine glue. For the permeable-barrier arenas, we used marine glue to seal the existing large holes and then drilled 15 evenly spaced 1/16′′ (0.16 cm) bit holes into the barrier; these holes let water flow through each side of the arena but limit eggs from passing from one side to the other. We then sealed the edges of the barrier to the inside of the aquaria. Glued aquaria were soaked in seawater for at least 12 h prior to being used in an experiment to ensure that the arenas were free of chemicals. To test the effectiveness of the permeable barrier in limiting eggs from passing through this barrier, we spawned *M. leidyi* overnight on one side of the arena (N = 7 arenas). We then calculated the percentage of eggs that passed from the occupied to the unoccupied side by the following morning. The solitary and no-barrier treatments used modified arenas without barriers.

### Animal collection and preparation

We collected 197 *M. leidyi* between July 28–August 23, 2016 and April 26–May 8, 2017 in Flagler Beach, Florida and transported them in buckets to the Whitney Laboratory for Marine Bioscience in St. Augustine, FL. We put each *M. leidyi* through a series of washes in 1 μm filtered and UV sterilized seawater to remove any substances (e.g., chemicals, particulate, etc.) that may have an influence on downstream behaviors and then placed them in individual bowls with 250 mL of seawater. We gave each *M. leidyi* a unique identification code and then measured the oral/aboral axis of every individual to the nearest mm with calipers.

Reproductive output positively correlates with body size in *M. leidyi* [30]. To control for the effect of size, we first sorted all *M. leidyi* by size from largest to smallest. We then grouped five *M. leidyi* together based on their size; for example, the five largest individuals were grouped together as were the five smallest. We refer to these groups as experimental blocks. Each block contained three treatments: one *M. leidyi* by itself in an undivided arena (“solitary” treatment; Fig. 1a), two *M. leidyi* in an undivided arena (“no-barrier arena” treatment; Fig. 1b), and two *M. leidyi* in an arena permeable by a divider (“permeable-barrier arena” treatment; Fig. 1c). We conducted a “sealed-barrier treatment” (Fig. 1d; described below) with the *M. leidyi* collected in 2017.

The permeable-barrier arena treatment acted as our experimental set-up to test for regulation of egg production. *Mnemiopsis leidyi* in the permeable-barrier arena cannot physically interact, which could be necessary for egg-regulating cues. To minimize this effect, and to more closely mimic the procedure from our original study [30], prior to being placed in the arena, *M. leidyi* in the no-barrier and permeable-barrier arenas were first placed with their paired partner for an hour in a 4′′ (10.2 cm) glass “interaction” bowl with 250 mL of filtered seawater. This set-up allows *M. leidyi* to physically interact before entry to the arena. We then used the water from these interaction bowls along with an additional 500 mL of UV sterilized and filtered seawater to fill the arena with a total of 750 mL seawater. Thus, any chemical cues exchanged during the 1 h of physical interaction should be present throughout the experiment. For the sealed-barrier treatment, we conducted the experiment as the permeable-barrier treatment described above with two exceptions. (1) Paired *M. leidyi* were isolated and never together in an interaction bowl before being placed in their separate sides of the arena, and (2) we did not drill holes in the barrier, thus blocking any water from moving between sides of the arena. Since *M. leidyi* did not interact or share water before or during this experiment, any potential signaling between pairs should be prevented.

For all treatments, we placed *M. leidyi* in the dark at 18:00 to spawn overnight [30]. At 9:00 the following morning, we removed each *M. leidyi* from their arenas and siphoned out the water from the arena into beakers. For the permeable and sealed-barrier arenas, we siphoned the water from each side of the arena simultaneously into separate beakers. We passed the siphoned water through a 70-μm filter to collect the eggs and then pipetted the eggs into a 2′′ (5 cm) diameter bowl [30]. After allowing time for the eggs to settle, we estimated the number of eggs in each bowl using the method described in Sasson and Ryan [30]. This estimation method reliably predicts to the total number of eggs spawned [30]. Thus, per block, we generated one count for the solitary and no-barrier arena treatments and two counts for the permeable-barrier arena (one per side of the arena). Two counts for each sealed-barrier arena (one per side) were made independent of these blocks. After counting, we tested the integrity of the sealed barrier’s seal in each arena to ensure that no water had passed across the divider during the experiment.

### Egg production and viability analyses

To compare the numbers of eggs spawned across treatments, we used a standard least squares model with treatment (“solitary”, “no-barrier arena”, and “permeable-barrier arena”) as the main effect, eggs spawned as the response variable, and block ID as a blocking effect. We then used Tukey’s HSD test to compare each treatment. We conducted an additional ANOVA test to compare egg production between the sealed-barrier treatment to the other three treatments. Egg counts for sealed and permeable-barrier experiments were determined by summing both sides of the barriers.

For each treatment, we counted the number of eggs showing signs of development 24 h after fertilization. We divided this count by the total number of eggs from the previous count to calculate offspring viability [30]. We used standard least squares model with treatment as the main effect, percent eggs developed as the response variable, and block ID as a blocking effect. We used Tukey’s HSD test to compare viability across treatments. We ran an additional ANOVA to compare viability from the sealed-barrier treatment to the other three treatments. We then used Tukey’s HSD test to compare viability across these four treatments.

The two least squares models were analyzed in JMP 12.0 (SAS, Cary, NC). Since individuals normally spawn hundreds of eggs, arenas with 25 or fewer total eggs were not included in the egg spawned or egg viability analyses [30].

### Gamete trading analyses

To test for gamete trading, we compared our data from the permeable and sealed-barrier arena experiments to each other as well as to a distribution of randomly generated trials. We calculated first the total number of eggs laid in each arena by combining the estimated number of eggs from both sides. We determined the proportion of eggs laid by each individual by dividing each side’s estimated count by the total. We then calculated the absolute difference in proportion of eggs laid across the two arena sides. We excluded from our analysis three permeable arena replicates in which neither individual spawned eggs (i.e. zero eggs on both sides). We performed this calculation for all replicates (N = 26 for both the permeable and sealed-barrier arena experiments) and then summed the absolute proportional differences across all replicates to create an absolute difference total for each treatment.

To test if the results from our sealed and permeable-barrier treatments were significantly different than each other, we ran a t-test to compare the absolute proportional differences between the permeable and sealed-barrier treatments.

To test if the results from our sealed and permeable-barrier treatments were significantly different than random, we created a distribution of 10,000 simulated absolute difference totals from randomly generated data. For each simulated replicate, a random number (a) between 0–1 was selected to represent the proportion of eggs on one side of the arena. We then subtracted this number from one to find the proportion of eggs on the other side of the simulated arena (b). We then calculated the absolute proportional difference between the two values (absolute value [a–b]) for 26 simulated replicates. We summed the absolute differences from those 26 replicates to create a simulated absolute difference total. We repeated this process 10,000 times to create a normal distribution of 10,000 simulated absolute difference totals. The Perl script used to create this distribution is available online (https://github.com/josephryan/2017_Sasson_and_Ryan).

We compared the absolute difference totals from our permeable and sealed-barrier arena data to the simulated distribution. An absolute difference total lower than 95% of the simulated totals (i.e. falling on the far left end of the distribution) would indicate that paired *M. leidyi* spawned statistically similar number of eggs on each side of the arena. An absolute difference total falling between the left and right tails of the distribution would suggest that the number of eggs produced by *M. leidyi* was random with respect to the number of eggs produced by the paired *M. leidyi*. An absolute difference total greater than 95% of the simulated values (i.e. falling on the tail-right end of the distribution) would indicate a significantly different number of eggs produced on the two sides of the arena. This last result is expected in permeable arenas if spawning behavior is regulated by the presence of conspecifics.

For the permeable-barrier arena experiment, we calculated a *p*-value for our result by dividing a count of the number of simulated absolute difference values larger than our permeable-barrier absolute difference value by 10,000 (the number of simulated replicates in the distribution). This calculation is a one-tailed test since, if spawning is coordinated, we expect our permeable-barrier absolute difference total to fall on the right side of the distribution. For the sealed-barrier arena, we calculated the *p*-value by dividing a count of the number of simulated difference values smaller than our sealed-barrier absolute difference total by 10,000. This calculation is also one-tailed test since we expect similar sized *M. leidyi* to spawn similar number of eggs in the absence of any coordination, thus falling on the left side of the distribution.

In two replicates of the permeable-barrier arena experiment, the paired individuals combined to have 25 or fewer total eggs. To ensure that our results were not significantly affected by these two cases, we removed these two replicates and then reran the t-test comparing the permeable and sealed-barrier arena results and repeated the procedure to compare the permeable-barrier absolute difference total to a newly generated random distribution (N = 24 for both the experimental and simulated replicates).

## Results

We collected a total of 145 *M. leidyi* for the experimental blocks; they ranged in sizes from 30 to 57 mm (mean: 38.8 ± 6.7 mm SD). Individuals were of relatively similar sizes within experimental blocks (mean standard deviation of sizes within each experimental block = 2.4 ± 2.1 mm). The 52 *M. leidyi* we collected for the sealed-barrier treatment ranged in size from 31 to 64 mm (mean: 42.5 ± 7.4 mm SD).

We showed that the barriers in the permeable-barrier arenas largely prevented eggs from drifting from one side of the arena to the other. When we placed *M. leidyi* on one side of a permeable-barrier arena and left the other side empty (N = 7), we found that only 2.3 ± 1.2% SD of the total eggs spawned overnight had drifted across the barrier by the following the morning.

**Fig. 2:**
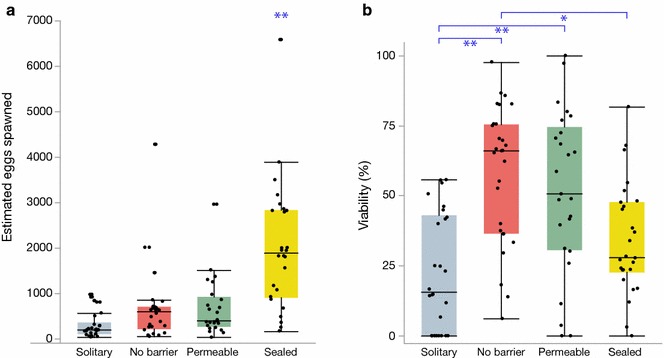
Egg numbers and viability. **a** Estimated number of eggs spawned across treatments. *Mnemiopsis leidyi* in the sealed-barrier arenas spawned significantly more total eggs than individuals in the other three arenas. **b** Significant differences in egg viability 24 h post spawn across arenas. *p < 0.001. **p < 0.0001

We found a significant difference in the number of eggs spawned across our initial three treatments (standard least squares, F_30,48_ = 4.0, p < 0.001). Solitary individuals in the undivided arenas (Fig. 1a) spawned significantly fewer eggs than either the sum of the two animals in the permeable (Tukey’s HSD test, p = 0.02) or no-barrier arenas (Tukey’s HSD test, p < 0.01). Permeable and no-barrier arenas (Fig. 1c, d) did not differ from each other in the number of eggs spawned (Tukey’s HSD test, p = 0.98). Within the model, we also found an effect of block ID on estimated egg numbers (p < 0. 00001), which was expected as blocks were designated by body size. Additionally, we found that individuals in sealed-barrier arenas spawned significantly more eggs than individuals in the other three arena treatments (Fig. 2a, ANOVA, F_3,101_ = 18.73, p < 0.0001). We found no difference in eggs spawned across the other three treatments; block effects were not considered in this analysis.

Our overall model comparing viability showed significant differences across treatments (standard least squares, F_30,48_ = 1.9, p = 0.02). The viability for *M. leidyi* spawning alone was lower than the viability of no-barrier (Tukey’s HSD test, p < 0.0001) and permeable-barrier *M. leidyi* pairs (Tukey’s HSD, p < 0.001). The viability of permeable and no-barrier arenas did not significantly differ (Tukey’s HSD, p = 0.57). We found no effect of block ID on viability in our model (p = 0.52). Additionally, we found significant differences in viability across all four treatments was significant (Fig. 2b; ANOVA, F_3,101_ = 12.79, p < 0.0001). Offspring in the sealed-barrier treatment had lower viability than the no-barrier treatment (p < 0.001) and the permeable-barrier treatment, although this latter result was not significant (p = 0.051). Viability in the sealed-barrier arenas did not differ from the viability of the solitary treatment (p = 0.28).

### Gamete trading

**Fig. 3:**
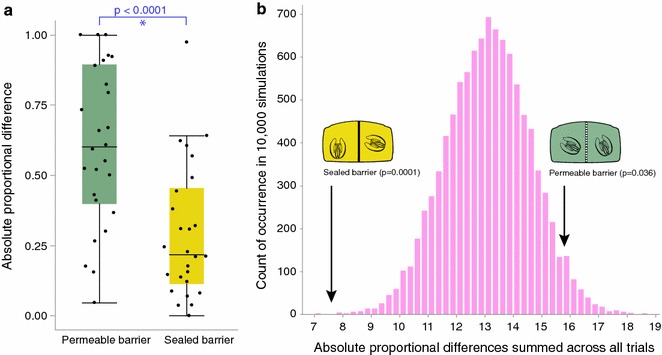
Evidence for communication. **a** Permeable and sealed-barrier comparison. We found that the permeable and sealed-barrier treatments significantly differed in their absolute proportional differences. **b** Distribution of simulated absolute values with 26 replicates. Arrows point to absolute value differences calculated from the permeable and sealed-barrier treatments. Both treatments significantly differed from random on opposing sides of the random distribution

To test for differences between the results of the permeable and sealed-barrier treatments, we performed a t-test. We found a significant difference between the absolute difference totals of these two experiments (Fig. 3a; N = 52, t-ratio = − 4.3, p < 0.0001). To further test if egg production of paired *M. leidyi* in the sealed and permeable-barrier treatments was non-random, we compared these results to a random distribution of absolute difference totals. We calculated an absolute difference total of 15.8 for permeable-barrier experiments, which was higher than all but 358 of the 10,000 simulated absolute totals (Fig. 3b; N = 26, p = 0.036). This result suggests a greater difference in the number of eggs on either side of the divided arenas than predicted by chance. We calculated an absolute difference total of 7.6 for the sealed-barrier treatment, which was lower than all but one simulated absolute value (Fig. 3b; N = 26, p = 0.0001). This result suggests that egg counts between pairs in this treatment were more similar than would be expected by chance.

We found 25 or fewer total eggs produced per arena in two replicates of the permeable-barrier treatment. To test if these two replicates were unduly influencing the results, we performed the analyses after removing these two results. We still found a significant difference between the permeable and sealed-barrier treatments after removing these replicates (N = 50, t-ratio = − 4.1, p = 0.0002). We also saw a similar trend when comparing the permeable-barrier experiment without those two replicates to a random distribution, although the result was not significant (N = 24, p = 0.066).

It is unclear whether seasonal difference affected our comparison of permeable and sealed barrier arenas. We attempted to explore this possibility by comparing egg output from sized-matched individuals from the solitary arenas to that from the permeable and sealed barrier experiments. Unfortunately, these comparisons involve small sample sizes and suffer from date of collection drawbacks as well. Nevertheless, these analyses are included as supplemental info. We believe that our use of relative egg numbers rather than absolute egg numbers mitigates most of the potential issues related to seasonality.

## Discussion

We found that *M. leidyi* of similar size on either side of a sealed-barrier arena produce highly similar numbers of eggs. In contrast, *M. leidyi* of similar size on either side of a permeable-barrier arena produce very different numbers of eggs. In both cases, we find these differences to be significantly different from random expectations. These results suggest that, when in contact with conspecifics, *M. leidyi* regulates egg production, perhaps to mitigate the costs of inbreeding depression associated with self-fertilization. The higher total egg production in the sealed-barrier arenas compared to the permeable and no-barrier arenas may further support the hypothesis that the detection of conspecifics results in the reduced egg output of one partner in spawning pairs. However, it cannot be ruled out that this difference in total egg production is due to a seasonal effect since the sealed-barrier experiments were conducted at a different time of year, or because the *M. leidyi* were slightly larger than in the other treatments. However, the increased egg production found in the sealed-barrier treatment is much higher than would be predicted based on body size alone [30]. Whatever the cause for this increase, we have no reason to think that seasonal differences in total egg production should affect the relative egg numbers on each side of the arena of paired *M. leidyi*.

Our data replicate previous findings showing that self-fertilization leads to decreased viability. We found significantly lower offspring viability in the solitary and sealed-barrier treatments compared to arenas where pairs could interact (Fig. 2b), providing additional support to the hypothesis that self-fertilization is costly in *M. leidyi* [30].

### Possible mechanism

The modulation of spawning behavior is likely a response to chemical cues, but the nature of these cues, where they are produced, and how they are received is unknown. *Mnemiopsis leidyi* continually releases mucus into the surrounding water, and it is know that this mucus can be detected by non-ctenophore species [35]. One possibility is that this mucus transports one or several signaling molecules that act as chemical signals used in conspecific communication.

Not much is known about chemoreception in ctenophores at the level of cells. Actin pegs on the lips of ctenophores and onion root cilia found on the lips of beroids and epidermis of other ctenophores have been implicated as perhaps having a chemosensory role [34, 36, 37]. These cells make synaptic contacts with gland cells and adjacent neurites [38]. Tamm and Tamm [34, 37] hypothesized that the actin pegs are mechanoreceptors and that the onion root cilia are chemosensitive. Similar structures have been described in the tentacles of cydippid ctenophores and the “fingers” of *Leucothea* [36, 39–41], but not in *M. leidyi*; however, Tamm [42, 43] observed bristle-like structures that project from the apical organ floor in *M. leidyi* and other ctenophores. In addition, *M. leidyi* has onion root cilia in the epidermis of the auricles. Despite not knowing the function of these bristles or the onion root cilia of the auricles, these are our current best guesses for *M. leidyi* structures involved in chemoreception.

Another possibility is that there is a mechanical component to the regulation of eggs. We have observed that, when in the presence of conspecifics, *M. leidyi* often pair up and stay in physical contact with each other for long periods of time. Whether this behavior occurs in nature and whether signaling occurs during this contact is unknown. In our permeable barrier trials, paired *M. leidyi* were placed together in the same bowl for an hour prior to being placed in the arenas, which allowed them the opportunity to physically interact. Although we separated individuals into their arenas for hours prior to spawning, this physical interaction may also have acted as another signal for the detection of conspecifics. Future field and laboratory studies into the effect of physical contact on reproductive behaviors in *M. leidyi* could prove insightful.

It remains unclear how paired *M. leidyi* decide which individual should spawn the majority of eggs. While egg numbers correlate with body size [30], the largest individual in each pair in our permeable arenas was not always the individual that spawned the most eggs. In fact, the smaller individual of the pair spawned the majority of eggs in eight of the 24 replicates where individuals differed in size. We did, however, try to reduce variation in *M. leidyi* size across pairs as much as possible; a clearer pattern might emerge if paired individuals differed greatly in size.

### Role of communication in situ

The locomotive abilities of *M. leidyi* are distinct from those animals where gamete trading has been described (i.e., annelids, fishes, and snails). Like many ctenophores, *M. leidyi* moves passively by the current with only a limited ability for directed movement via macrocilia. Thus the likelihood of two individuals spawning multiple times within proximity of each other is rare, making a reciprocal exchange of gametes unlikely.

*Mnemiopsis leidyi* are commonly found as both solitary swimmers as well as in dense “raft” populations [44] and so, the ability to modulate spawning at different density levels could be beneficial. At high densities, there may be a fitness advantage to limiting self-fertilization in the form of increased offspring viability (Fig. 2b) by spawning (mostly) one gamete type during a given mating bout. However, at low densities, it may be beneficial to spawn both eggs and sperm to boost total reproductive output despite the risk of inbreeding depression. Thus, the ability to modulate reproductive output in the presence of conspecifics may explain the maintenance of hermaphroditism in systems such as *M. leidyi* where inbreeding depression is costly. This same behavior may also provide insights into how the transition from hermaphroditism to separate sexes may occur in animals.

### Is this behavior gamete trading?

One essential aspect of gamete trading is that individuals in mating pairs reciprocally alternate between gamete type; unfortunately, we are unable to consistently spawn *M. leidyi* more than once (due to reduced nutritional intake while in the lab), and we are therefore unable to reliably test the reciprocal aspect of gamete trading. Sperm would also be expected to be differentially released in a gamete trading scenario, but we did not compare sperm production across sides of the barriers. The increased egg viability in the permeable barrier arenas suggests that sperm traveled between sides of the permeable barrier arenas, which would have made it difficult to assign sperm production to specific individuals. Furthermore, the amount of water in each arena diluted any sperm to such a degree to make reliable counting of sperm difficult. Initial attempts to scan for sperm in sampled water proved ineffective. Thus, while our experiments produce results consistent with what would be expected if gamete trading were occurring, we cannot say that this behavior definitely qualifies as gamete trading per se.

## Conclusion

In this study, we show that ctenophores modulate spawning behavior in the presence of conspecifics perhaps via chemical cues, possibly to limit self-fertilization. To our knowledge, this study provides the first evidence of conspecific signaling in Ctenophora. Moreover, the data show that spawning behavior involves coordination with other individuals. Such behavior requires complex signaling between individual ctenophores that has not heretofore been observed and may be surprising in an animal lineage that diverged from the rest of animals early in the history of animal evolution. Understanding how the mechanisms of communication in ctenophores compares to other animal lineages may provide insight into the evolution of communication in Metazoa.

This report of coordinated behavior in a species of ctenophore presents a whole new set of questions outside of mechanism. Does this behavior occur in other ctenophores? What role does this communication have on *M. leidyi’s* ability to establish in non-native regions? Is this communication evolutionary important? It is intriguing to realize that the answers to these and other exciting questions are now answerable. Future studies into the mechanism, dynamics, evolution, and role of this behavior in situ should lead to fascinating insights into the biology of ctenophores.

An understanding of chemical cues in ctenophores would not only bolster our nascent understanding of ctenophore signaling and communication, but also provide valuable insights into the broader question of the evolution of sensory systems across animals. This study suggests the possibility that early animals had a similar ability to use complex communication to maximize reproductive efficiency.

## Additional file

**Additional file 1.** Comparison of solitary arenas to permeable and sealed barrier arenas.
