# Supplementary material for: The ctenophore Mnemiopsis leidyi regulates egg production via conspecific communication
Source: BMC Ecol. 2018 Mar 26;18:12. doi: 10.1186/s12898-018-0169-9 (PMC5868061; doi:10.1186/s12898-018-0169-9)
Supplement: Supplementary file 1 — Additional file 1. Comparison of solitary arenas to permeable and sealed barrier arenas. [file 12898_2018_169_MOESM1_ESM.docx]

**Comparison of solitary arenas to permeable and sealed barrier arenas**

In an effort to explore possible effects of season difference in our comparison of permeable and sealed barrier arenas, we compared these treatments to the egg outputs from individuals from the solitary arenas. To do this, we created pairs of solitary arena individuals to treat as if they were in the sealed barrier experiment. We sized-matched individuals as closely as possible to create pairs of solitary individuals. We then took the absolute proportional difference between these matched pairs as we had done with the permeable and sealed barrier arenas. Finally, we compared the absolute proportional differences from the solitary matched pairs (N =14) to the absolute proportional differences from the permeable and sealed barrier arenas (N = 26 for each) using ANOVA. Post-hoc comparisons were done with Tukey’s HSD test.

**Results**

The overall ANOVA (F_2,63_ = 7.8, p < 0.001) was significant, but this was due to a significant difference between the permeable and sealed barrier arenas (Tukey’s HSD, p = < 0.001). The absolute proportional differences from the solitary matched pairs (mean = 0.43 ± 0.37 SD) was lower than but not significantly different from the permeable barrier arenas (mean = 0.61 ± 0.28 SD, Tukey’s HSD, p = 0.18) and higher than but also not significantly different from the sealed barrier arenas (0.29 ± 0.24 SD, Tukey’s HSD, p = 0.30).

**Discussion**

We performed these analyses in response to a reviewer’s inquiry into the effect of seasonal differences. We recognized weaknesses in these comparisons prior to running these analyses, but agreed with the reviewer that these analyses might be helpful to readers. We did not find that the solitary matched pairs differed from the permeable barrier arenas, as might be expected under the assumption that the egg output of solitary individuals should follow the same patterns for matched individuals in the sealed barrier arenas. However, conducting such a post-hoc pairing of solitary individuals leads to its own problems. While we sized matched individuals from the solitary arenas for this comparison, only three of the resultant 14 paired *M. leidyi* were collected on the same day and five of the paired matches were collected more than a week apart. Previous research has shown that differences in nutrition play a large role in determining *M. leidyi* egg output (e.g. Jaspers et al. 2015), and limited food over just one day can affect reproductive output (personal observations). Thus, the majority of paired individuals may have experienced vastly different nutritional environments prior to collection, a problem not found in the permeable and sealed barrier arenas where all matched individuals were collected together. This issue plus the reduced sample size make a direct comparison between our original sealed barrier experiments and these new analyses difficult to interpret.
